# Development and implementation of the National Heart, Lung, and Blood Institute COVID-19 common data elements

**DOI:** 10.1017/cts.2022.466

**Published:** 2022-09-26

**Authors:** Alexandra Weissman, Alex Cheng, Alex Mainor, Elizabeth Gimbel, Kayla Nowak, Huaqin (Helen) Pan, Jeran Stratford, Alyssa Merkel, Caroline Taylor, Heather Meier, Jeanette Auman, Tracy L. Nolen, Christopher J. Lindsell, David T. Huang

**Affiliations:** 1 Department of Emergency Medicine, University of Pittsburgh School of Medicine, Pittsburgh, PA, USA; 2 Vanderbilt University Medical Center. Nashville, TN, USA; 3 Department of Critical Care Medicine, University of Pittsburgh School of Medicine, Pittsburgh, PA, USA; 4 RTI International, Research Triangle Park, NC, USA

**Keywords:** Common data element, CDE, clinical trials, COVID-19, FAIR, CONNECTS

## Abstract

**Background:**

Coronavirus Disease 2019 (COVID-19) instigated a flurry of clinical research activity. The unprecedented pace with which trials were launched left an early void in data standardization, limiting the potential for subsequent data pooling. To facilitate data standardization across emerging studies, the National Heart, Lung, and Blood Institute (NHLBI) charged two groups with harmonizing data collection, and these groups collaborated to create a concise set of COVID-19 Common Data Elements (CDEs) for clinical research.

**Methods:**

Our iterative approach followed three guiding principles: 1) draw from existing multi-center COVID-19 clinical trials as precedents, 2) incorporate existing data elements and data standards whenever possible, and 3) alignment to data standards that facilitate data sharing and regulatory submission. We also supported rapid implementation of the CDEs in NHLBI-funded studies and iteratively refined the CDEs based on feedback from those study teams

**Results:**

The NHLBI COVID-19 CDEs are publicly available and being used for current COVID-19 clinical trials. CDEs are organized into domains, and each data element is classified within a three-tiered prioritization system. The CDE manual is hosted publicly at https://nhlbi-connects.org/common_data_elements with an accompanying data dictionary and implementation guidance.

**Conclusions:**

The NHLBI COVID-19 CDEs are designed to aid data harmonization across studies to achieve the benefits of pooled analyses. We found that organizing CDE development around our three guiding principles focused our efforts and allowed us to adapt as COVID-19 knowledge advanced. As these CDEs continue to evolve, they could be generalized for use in other acute respiratory illnesses.

## Introduction

The Coronavirus Disease 2019 (COVID-19) pandemic rapidly generated an immense amount of study globally. There are over 8,000 studies listed on ClinicalTrials.gov that have tested or are actively testing a diverse range of interventions [1]. Rapid study initiation resulted in the earliest studies selecting their own covariates and measurement methods, even within the same data element [2]. Lack of standardized data elements makes comparing observations and interventions across studies difficult and hinders reproducibility and generalizability. One standardization approach is using clearly defined variables with specific response values common to multiple data sets across different studies and is termed common data elements (CDEs) [3–6]. CDEs can be used in a variety of study designs to reduce the burden of creating new case report forms (CRFs), increase homogeneity of variables and outcome measures, and enable comparisons across studies such as meta-analyses [3–5]. Finally, CDEs can enable data interoperability in accordance with the FAIR principles (Findable, Accessible, Interoperable, Reusable) of scientific data management [7].

In late March 2020, the National Heart, Lung, and Blood Institute (NHLBI) funded researchers at the University of Pittsburgh to develop COVID-19 specific CDEs that could be employed in NHLBI-sponsored COVID-19 research. Separately, on June 15, 2020, the NHLBI created the Collaborating Network of Networks for Evaluating COVID-19 and Therapeutic Strategies (CONNECTS) to bring together existing clinical trial networks into a more formal “network of networks.” The primary mission of CONNECTS was to swiftly design and implement large multi-center COVID-19 trials across acute care networks. To maximize data sharing and reuse, CONNECTS included a Data Harmonization Core (DHC) comprising members from RTI International (the CONNECTS Administrative Coordinating Center) and Vanderbilt University Medical Center (VUMC, the CONNECTS Administrative Coordinating Center Science Core). The University of Pittsburgh CDE team joined with the CONNECTS DHC to rapidly develop a concise set of CDEs for immediate use in NHLBI-sponsored interventional trials in both outpatient and inpatient COVID-19 populations.

Herein, we detail the development processes, organization, initial implementation, evolution, and projected use of the NHLBI COVID-19 CDEs. This publication aims to provide rationale for CDE design for COVID-19 clinical trialists as well as lessons learned to guide future CDE development efforts.

## Methods

At project inception, there were no published COVID-19 specific CDEs available, and few existing National Institutes of Health (NIH) CDEs that specifically addressed clinical trials targeted toward respiratory infections in ways suitable for use in NHLBI COVID-19 trials [8]. Therefore, we developed a set of COVID-19 CDEs following three principles:

1) Draw from existing multicenter COVID-19 clinical trials as precedents.

2) Incorporate existing data elements and data standards whenever possible.

3) Alignment to data standards that facilitate data sharing and regulatory submission.

The partnership between the CONNECTS DHC and the University of Pittsburgh CDE team brought together experts with complementary backgrounds and clinical trial expertise to design CDEs that can serve multiple audiences. The University of Pittsburgh team consisted of an emergency medicine physician, a critical care physician with an emergency medicine background, and a project manager. The CONNECTS team included two biostatisticians, three informaticians, and a project manager.

The University of Pittsburgh team liaised with leaders from the NHLBI-sponsored Prevention and Early Treatment of Acute Lung Injury (PETAL) [9] consortium and Strategies to Innovate Emergency Care Clinical Trials Network (SIREN) [10], as well as with an expert from the GCS-NeuroCOVID group [11], to obtain feedback from critical care, emergency medicine, and neurology clinical researchers launching COVID-19 trials and cohort studies. The CONNECTS DHC obtained feedback from other CONNECTS stakeholder groups, including those responsible for master platform trial design. Through an iterative process, the initial drafts were restructured and data elements were refined, classified, and aligned to existing data standards whenever feasible (Supplementary Table 1). This process occurred through weekly to bimonthly teleconference meetings starting in August 2020 (Supplementary Figure 1).

Data elements were organized and developed by specific domains including demographics, medical history, symptoms, and treatments. Description of the different domains as well as guidance for implementing the data elements into study CRFs can be found in the published common data elements manual (CDEM) [https://nhlbi-connects.org/common_data_elements]. The CDEM has an accompanying data dictionary with detailed information about the data elements. The CDEs are intended as a living resource to be continually updated and refined and were approved by the NHLBI CONNECTS Steering Committee.

## COVID-19 Trial Precedents (Principle 1)

CDE development built upon precedent gleaned from CRFs and Manuals of Procedures for existing multicenter national and international COVID-19 studies and trials (Supplementary Table 2). Iterative CDE revision was continuously driven by review of newly available study documentation of registries, observational trials, and interventional trials. Initial formatting of the CDEs, including question format as well as response formats and options, was selected based on prevalence across all studies reviewed, existing data standards, and the initial intended application of the CDEs to clinical trials supported by NHLBI. Definitions for CDEs, and the content of CDEs themselves, were developed in line with existing CRFs and modified through several rounds of revision and input from CONNECTS members including research nurses, clinical trialists, emergency medicine, and critical care physicians to ensure that CDEs could be practical as well as comprehensive. This process resulted in the recommendation of a tiered approach to CDEs, where the CDEs are classified as:

1. **Core** to interpreting a study, such as critical baseline characteristics, inclusion/exclusion criteria, study interventions, and main outcomes.
2. **Preferred** to fully characterize a clinical study cohort, describe differential treatment effects, and maximize overlap among study data sets for participant-level data meta-analyses.
3. **Optional** to provide additional clinical insight while still incorporating operational definitions and a standardized format for collection in cases where these data are collected.

## Incorporate Existing Data Elements and Data Standards (Principle 2)

To avoid “reinventing the wheel” [3], we incorporated relevant CDEs from existing sources whenever possible. We queried the NIH CDE Repository [8] for existing CDEs pertinent to COVID-19 research to date and found that the majority of respiratory disease CDEs were appropriate for epidemiologic assessments, but not tailored to clinical trials. Many of the existing NIH CDEs for respiratory diseases focused on respiratory mechanics that surpassed the level of detail necessary for COVID-19 trials, while others were not pertinent to COVID-19 trials (e.g., pulmonary function testing, ventilator recruitment techniques, polysomnography assessments). We adapted NIH CDEs describing oxygen delivery and respiratory rate to fit with precedents already in use in several COVID-19 trials. CDEs related to demographics and social determinants of health currently in use by the *All of* Us Research Program [12] and the PhenX Toolkit [13] were incorporated to address the increasing focus on health disparities in biomedical research and highlighted by the COVID-19 pandemic. Additionally, we developed *de novo* CDEs to cover social topics specific to COVID-19 (e.g., ability to self-isolate).

Relevant clinical variables from the Clinical Data Interchange Standards Consortium (CDISC) [14] Clinical Data Acquisition Standards Harmonization (CDASH) model [15] and Study Data Tabulation Model (SDTM) [16] were also included. The original Charlson Comorbidity Index [17] variables formed the basis of comorbidity selection and were expanded to include additional comorbidities pertinent to COVID-19 interventions and pathophysiology. Common severity scoring systems such as the Sequential Organ Failure Assessment (SOFA) [18] were reviewed to inform how relevant clinical and laboratory data might be used or interpreted, and thus the context and format with which they should be collected. In addition, international surveys regarding COVID-19 outcomes of interest were reviewed to identify additional variables of potential interest [19,20]. Concomitant medications of interest were compiled from precedent COVID-19 studies (Supplementary Table 2). Outcomes were initially informed by COVID-19 clinical trial precedents and the World Health Organization’s recommended minimal outcome dataset [21]. Finally, we reviewed the Common Terminology Criteria for Adverse Events (CTCAE) Version 5.0 definitions for defining and grading adverse events [22].

The demographics domain serves as an example of how we relied on existing standards to develop a subject area domain. Using the review processes outlined above, we identified numerous sources for variables to collect demographic information, including CDASH, the *All of Us* Research Program, PhenX, and ongoing COVID-19 study CRFs. We encountered a critical decision point early in this process – many of these sources collected similar demographic information with different questions or response options. We chose CDASH as our data standard largely due to the potential need to facilitate FDA submissions.

We applied our three-tier data prioritization system for each CDE by considering the level of desired specificity we could obtain weighed against important practical considerations. For example, a useful question for research purposes (e.g., household income), could be considered sensitive by some patients, potentially hindering their willingness to participate in research. Key information related to location (e.g., 9-digit ZIP code) could be invaluable for future analyses but raise privacy concerns regarding personally identifiable information and how it is collected, transmitted, stored, and used. For each CDE, we discussed and balanced each of these considerations when setting priority tier levels. This process allowed us to create a robust set of demographics CDEs with specific considerations for the clinical trials space.

## Alignment to Data Standards that Facilitate Data Sharing and Regulatory Submission (Principle 3)

Data standards facilitate cross-study comparisons and analyses, and submission to regulatory authorities. Since many COVID-19 trials are clincal drug trials, we sought to make our CDEs compatible with CDISC. The CDISC organization develops and manages standards for clinical trial data and metadata collection, formatting, and submission to regulatory authorities [14]. As such, CDISC models served as an excellent framework for harmonizing data across varying study designs, therapeutics, and indications. As an added benefit, CDISC alignment facilitates regulatory data submission for any studies seeking regulatory approval. CDISC models also apply controlled terminology from the National Cancer Institute Thesaurus (NCIt) to ensure consistent data interpretation (e.g., all unit measurements apply controlled terminology from NCIt codelist C71620). NCIt terminology links to other terminologies such as SNOMED (Systemized Nomenclature of Medicine – Clinical Terms), LOINC (Logical Observation Identifiers Names and Codes), RxNORM, ICD (International Classification of Diseases), and CPT (Current Procedural Technology) in the NLM UMLS (National Library of Medicine Unified Medical Language System) Metathesaurus. The linkage from CDISC to NCIt terminology allows our CDEs to be readily translated to other common structures for pooled analyses and/or regulatory submissions.

The CDEs were designed based on CDISC standard domains, data structures, variables, and controlled terminology wherever feasible. Data elements and associated information required by CDISC were classified as “core” regardless of stakeholder feedback. Although CDISC served as an excellent starting point for our CDEs, only a few CONNECTS studies require regulatory data submissions at this time. By having CDISC-targeted CDEs instead of fully CDISC-compliant CDEs, we optimized our CDEs for data collection and analysis, while serving as an intermediate step towards full CDISC compliance. For COVID-19-specific questionnaires, we used custom findings domains organized by topic to streamline data harmonization and ensure data are readily accessible to analysts. For general domains, nonunique to COVID-19, we often used the CDISC domain directly (e.g., Adverse Events, Concomitant Medications, Healthcare Encounters, Laboratory Test Results). Occasionally, the CDISC structure was not directly beneficial for analysts, and we opted for a modified structure. We used a wide data structure because it streamlined the capture of detailed baseline measures that would have been reported in separate domains under CDISC (e.g., Substance Use). For example, CDISC’s Medical History domain is tall, with one record per question per subject; however, we used a wide Medical History domain, with one record per subject and one variable per question. This transposition was possible because we specified a fixed set of recommended medical history questions, and we did not need to accommodate other, study-specific questions.

In cases where we intentionally deviated from CDISC standards, we applied variable name fragments and collection methods motivated by CDISC standards. For studies seeking CDSIC compliant data submissions, these design choices faciliate mapping the NHLBI COVID-19 CDEs to CDISC standards.

## Implementation and Harmonization

In addition to CDE development, our team was charged with rapid implementation and dissemination of those CDEs to CONNECTS studies and COVID-19 researchers at large. CDE implementation during initial study design and CRF creation is preferable to harmonizing study data to CDEs following data collection. However, *post-hoc* harmonization is often needed for ongoing studies and enables a comparison across studies wherever study variables and CDEs overlap. Because several CONNECTS studies were already recruiting participants or had approved CRFs when we began CDE development, we provided these studies with the support to perform *post-hoc* harmonization.

**Fig. 1.**
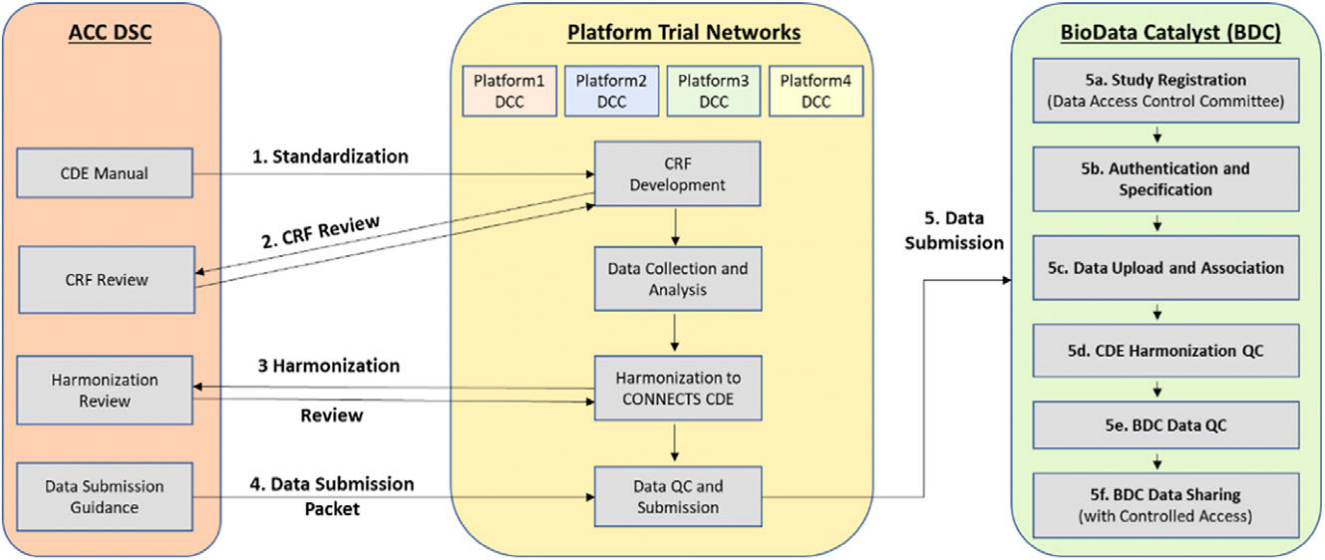
Overview of the CONNECTS data harmonization process. Legend: Administrative Coordinating Center (ACC); Data Standards Core (DSC); Data Coordinating Center (DCC); Common Data Element (CDE); Case Report Form (CRF); Quality Control (QC); CONNECTS (Collaborating Network of Networks for Evaluating COVID-19 and Therapeutic Strategies); BioData Catalyst (BDC).

Through the data harmonization process, we worked with study teams to map existing study variables to the NHLBI COVID-19 CDEs creating standardized datasets with common elements and structure (Fig. 1).

To begin the harmonization process, the study team compared its collected variables to the NHLBI COVID-19 CDEs. The team then assigned a “mapping level” indicating how well a study variable aligned to a specific CDE. These mapping levels included four response options – “identical” (the study team collected the data in the exact way recommended by the CDE), “comparable” (conceptually like CDE, but slightly different phrasing or response options), “related” (covers similar topic to CDE, but mapping relationship is uncertain), and “not mappable” (no study variable maps to the CDE). We reviewed these mappings and met with study teams to discuss questions and proposed revisions, with particular focus on developing solutions to improve mapping of “comparable” and “related” study variables to the CDEs. We then drafted harmonization instructions for each variable to be used by study team programmers or analysts to create final harmonized datasets.

Study teams were asked to submit a dataset that was transformed to conform to the NHLBI COVID-19 CDEs, and the associated harmonization document, as part of the study’s submission to BioData Catalyst [23]. This allows future researchers to find variables of interest in the *post-hoc* harmonized datasets.

## Results

**Fig. 2.**
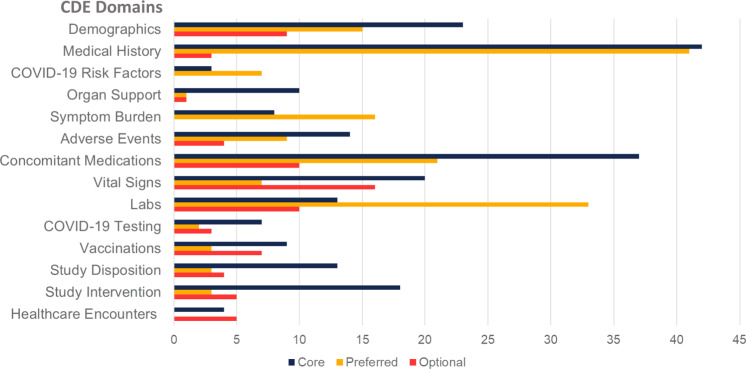
Number of common data elements by subject matter domain and requirement level. Legend: Common Data Element (CDE).

On completion, Version 1.0 of the unified CDEs, CDEM, and data dictionary was presented to senior investigators from NHLBI and the CONNECTS Steering Committee for review, revision, and final approval on December 16, 2020. Subsequently, we have updated the CDEs to reflect new knowledge (e.g., vaccination) and based upon feedback from studies during harmonization. The iterative development process produced a comprehensive set of CDEs arranged according to visit type (e.g., inpatient versus outpatient, baseline visit versus follow-up) and data element domain (e.g., demographics, medical history, vital signs, laboratory studies) and compiled into the CDEM (Fig. 2, Supplementary Table 3).

The accompanying data dictionary describes the element, variable name, variable label, variable type, length, CRF example question text and response options, and implementation notes; key terms and concepts are summarized in Table 1.

**Table 1.** Key terms and concepts for the National Heart, Lung, and Blood Institute (NHLBI) COVID-19 Common Data Element (CDE) Data Dictionary

Term/Concept	Description
Domain	Subject matter grouping for collection of a specific set of data elements (e.g., demographics, medical history). The domain abbreviation (tab name) is also the dataset name.
Element	Name, as specified in Data Dictionary
Variable	Variable name
CRF Reference	Linkage to corresponding variable in example case report forms (CRFs) available to all studies
Requirement Level	CDEs labeled as “Core” are required for NHLBI-CONNECTS funded trials. Those labeled as “Preferred” are strongly encouraged to be included. “Optional” CDEs are listed to ensure response options and formatting are consistent among studies that decide to collect those CDEs.
Study Type (IP, OP, D, N)	CDE requirement levels differ depending on the type of COVID-19 therapeutic study. In the data dictionary, recommendation levels will be followed by a combination of letters signifying the trials for which that recommendation is expected to apply: • IP: Admitted patient study • OP: Outpatient or post-discharge study • D: Drug intervention • N: Non-drug intervention
Variable Type	Type of variable to inform collection and programing (e.g., character, numerical).
Question	Suggested question language for collection of element in a CRF or questionnaire.
Response Options	Response options or derivation instructions for element collection.
Implementation Notes	Additional guidance around implementation, derivation, or programing logic.
BioData Catalyst (BDC) ID	Standardized variable name reference for upload to the BioData Catalyst data repository.

The CDEM describes how investigators can implement the CDEs and offers recommendations for consistent timing and frequency of data collection, among other guidance. Both the data dictionary and CDEM are posted to the CONNECTS public portal for ease of access for other researchers [24]. The Organ Support Domain CDEs are also in submission to the NIH CDE Repository for further dissemination and for easy importing of CRF data elements through the NIH CDE Repository application programing interface [25].

**Fig. 3.**
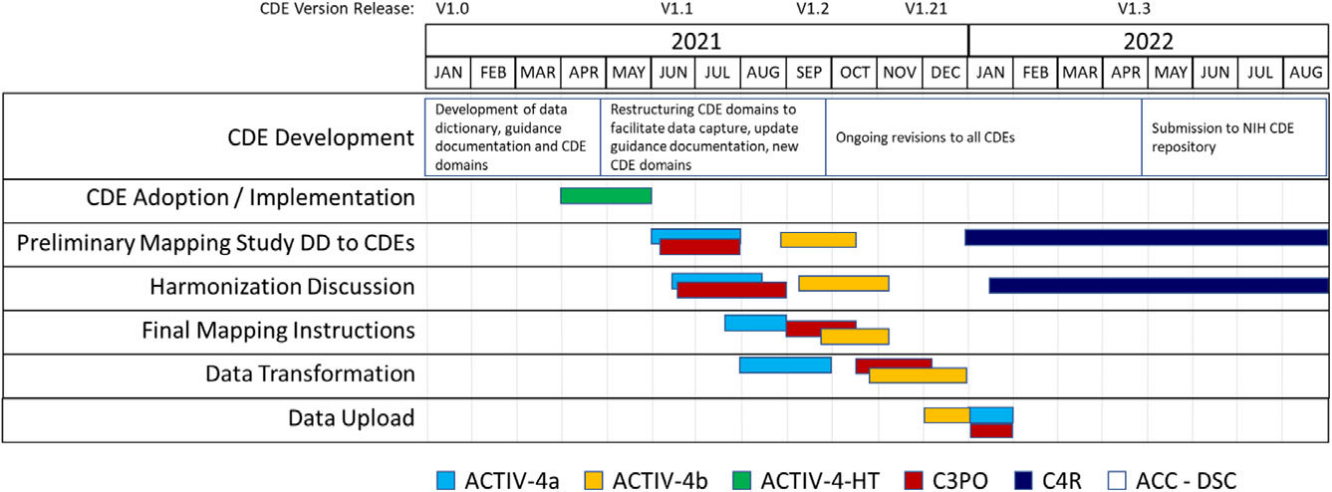
CDE implementation and harmonization activities. Legend: Accelerating COVID-19 Therapeutic Interventions and Vaccines Inpatient Trial (ACTIV-4a); Accelerating COVID-19 Therapeutic Interventions and Vaccines Outpatient Trial (ACTIV-4b); Accelerating COVID-19 Therapeutic Interventions and Vaccines Host Tissue (ACTIV-HT); Clinical Trial of COVID-19 Convalescent Plasma in Outpatients (C3PO); Collaborative Cohort of Cohorts for COVID-19 Research (C4R); Administrative Coordinating Center (ACC); Data Coordinating Center (DCC).

We began initial implementation activities in 2021 (Fig. 3). Accelerating COVID-19 Therapeutic Interventions and Vaccines (ACTIV)-4a Inpatient Trial [26,27] was the first study to complete study harmonization and uploaded their study data to BioData Catalyst in October 2021. The ACTIV-4b Outpatient Trial [28] was the second study to complete harmonization efforts and their study data was uploaded to BioData Catalyst in November 2021. The Clinical Trial of COVID-19 Convalescent Plasma in Outpatients (C3PO) [29] submitted their data in December 2021. Data harmonization efforts for the observational cohort study, C4R: Collaborative Cohort of Cohorts for COVID-19 Research, is under discussion in 2022 (Supplementary Figure 2).

As new studies have come online, adoption of the NHLBI COVID-19 CDEs is increasing. One study, ACTIV-4-Host Tissue [30], launched in June 2021, was able to standardize their data collection to the NHLBI COVID-19 CDEs at study design and CRF creation. Study personnel used the NHLBI COVID-19 CDE data dictionary and manual to create their initial study database within 2 weeks, adding and modifying only certain study specific and administrative data fields. ACTIV-4-Host Tissue data will be compatible with future studies that conform to the NHLBI COVID-19 CDEs. ACTIV-6 [31], while not sponsored by NHLBI, also adopted many of the NHLBI COVID-19 CDEs with the purpose of streamlining data pooling and reuse activities. We anticipate these studies will serve as models for future COVID-19 studies seeking to implement our CDEs.

## Results of Harmonization Efforts

**Fig. 4.**
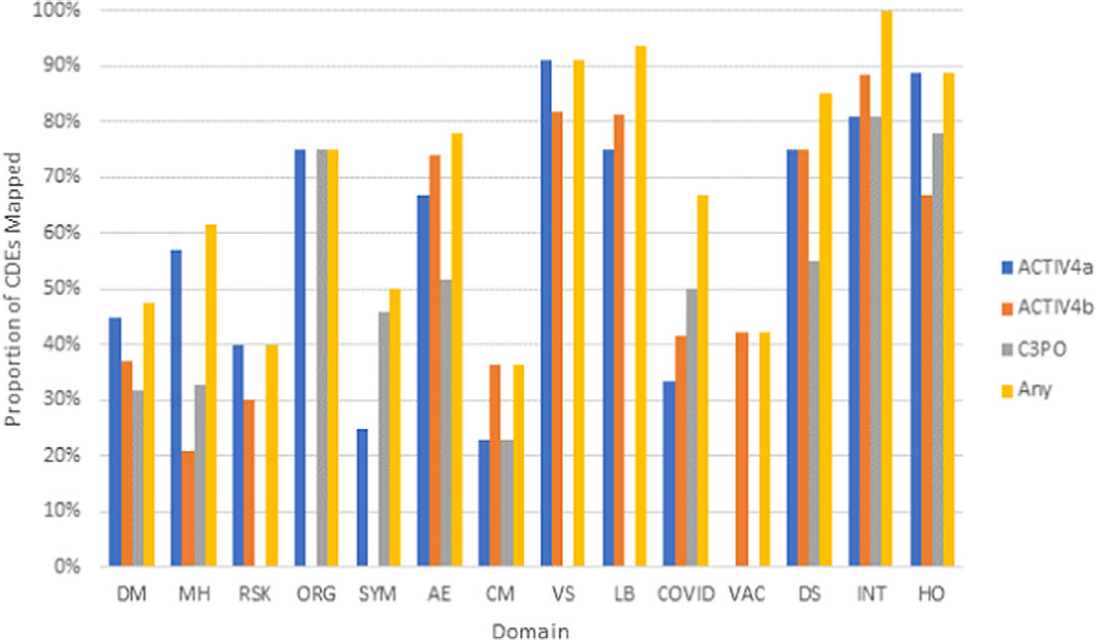
Proportion of CDEs populated with harmonized data by domain across the ACTIV-4a, ACTIV-4b, and C3PO studies. Legend: Domains are listed along the *x*-axis. Domain names: DM (Demographics), MH (Medical History), RSK (COVID-19 Risk Factors), ORG (Organ Support), SYM (Symptom Burden), AE (Adverse Events), CM (Concomitant Medications), VS (Vital Signs), LB (Clinical Labs), COVID (COVID-19 Testing), VAC (Vaccinations), DS (Disposition), INT (Intervention Exposure), HO (Healthcare Outcomes).

Three existing clinical studies completed retrospective harmonization to the NHLBI COVID-19 CDEs. Analysis of *post-hoc* harmonized datasets revealed that a majority of the study variables for ACTIV-4a (Supplementary Figure 3), ACTIV-4b (Supplementary Figure 4), and C3PO (Supplementary Figure 5) mapped to the CONNECTS CDEs, with greater than 70% of variables mapping to Organ Support, Vital Signs, Labs, Study Disposition, Study Intervention, and Healthcare Encounter CDE domains (Fig. 4). On average, each study mapped study-specific variables to 44% of all NHLBI COVID-19 CDEs. While certain domains appeared to be universal across studies (adverse events, study disposition, intervention, and healthcare encounters), the study type (in-patient vs. out-patient, drug intervention vs. nondrug intervention) also dictated whether any relevant study data was available for a given domain. This information may guide future revisions of the NHLBI COVID-19 CDEs.

**Fig. 5.**
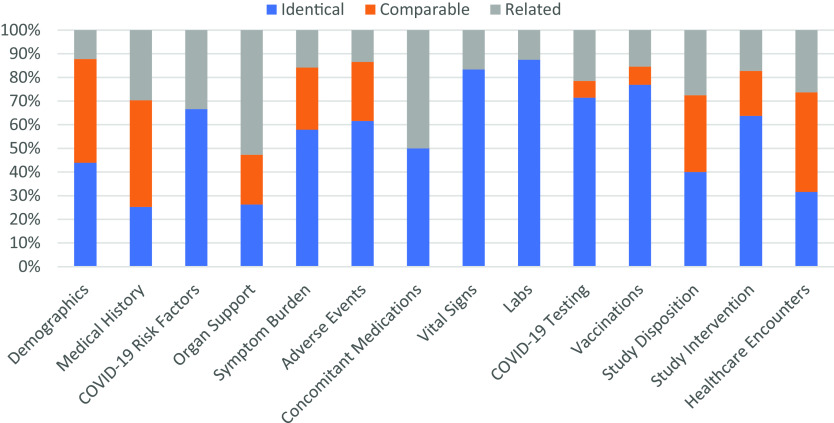
Proportion of common data elements (CDEs) at each mapping level that mapped to ACTIV-4a, ACTIV-4b, and C3PO in aggregate. Legend: The proportion of mapping levels assigned to the study variable(s)/CDE pairing across all three harmonized studies was evaluated and visualized for each CDE domain separately. An “Identical” mapping (blue) signifies study data was collected exactly as recommended by the NHLBI COVID-19 CDE. A “Comparable” mapping (orange) means that the study variable and NHLBI COVID-19 CDE are conceptually similar but differ in phrasing or response options. A “Related” mapping (gray) indicates that the study variable and the NHLBI COVDI-19 CDE covers a similar topic, but the mapping relationship is uncertain.

Across all three *post-hoc* harmonized studies, the mapping of study variables to relevant CDEs occurred most frequently at a mapping level of “identical” (49%) contrasted to “related” (23%) or “comparable” (28%). CDEs for Vital Signs, Clinical Labs, Intervention Exposure, and Vaccinations had the highest proportion of “identical” mapped variables based upon a comparison of mapping levels in mapped CDEs (Fig. 5).

Variables for Vital Signs, Clinical Labs, Disposition, Intervention Exposure, and Healthcare Encounters also mapped well regardless of inpatient or outpatient study type (Supplementary Figure 6). Within specific domains, the proportion of mapping levels assigned to CDEs were variable. Domains such as Demographics and Medical History have the most percentage of “comparable” and “related” mapping levels.

Of the studies that performed *post-hoc* harmonization, ACTIV-4a had the highest proportion of mapped variables (Supplementary Figure 4). “Identical” and “comparable” mapping levels were most common among CDEs recommended for inpatient interventional studies, such as Vital Signs, Clinical Labs, Healthcare Outcomes, and Organ Support. For the outpatient interventional trials, ACTIV-4b and C3PO, variables within the Adverse Events, COVID-19 Testing, Intervention Exposure, Disposition, and Healthcare Outcomes domains mapped well to the NHLBI COVID-19 CDEs (Supplementary Figures 4 and 5). ACTIV-4b did not collect data on Symptom Burden and Organ Support, while C3PO did not collect data for Vital Signs, Clinical Labs, and COVID-19 Risk Factors. The abundance or absence of inpatient/outpatient CDEs is consistent with the inpatient/outpatient settings of the three studies.

## Discussion

Creating harmonized datasets enables data pooling and can strengthen subgroup analyses and generalizability while characterizing sources of variation. With NHLBI sponsorship, CONNECTS Steering Committee oversight, and input from multiple stakeholders, we synthesized a wide array of information to develop a concise yet comprehensive set of CDEs for clinical studies of COVID-19. Our charge was to rapidly develop and implement CDEs applicable to a wide range of COVID-19 clinical studies, even while knowledge about COVID-19 was being generated. We engaged in serial review and refinement over the first year of the pandemic to develop the initial CDEs with a data dictionary and implementation manual. Subsequently we have continued this iterative process to update the CDE versions in real time. After supporting the harmonization process, our team also facilitated upload of all harmonized datasets to BioData Catalyst. We additionally supported the breadth of data upload activities from data standardization and CRF review through technical requirements for data upload and submission. The NHLBI COVID-19 CDEs have now been adopted by prospective studies (ACTIV-4 Host Tissue), harmonized for completed studies (ACTIV-4a phase 1, ACTIV-4b, C3PO), and will be harmonized for ongoing studies (ACTIV-4a phase 2, ACTIV-4c). These harmonized data sets can be pooled for specific hypothesis testing outside the scope of the original designs of each individual study or for future meta-analyses of COVID-19 interventions beyond CONNECTS. We acknowledge that as the NHLBI COVID-19 CDEs developed, other NIH CDE efforts were underway, such as the more demographic and social determinates of health-focused NIH RADx-UP CDEs led by the Duke Clinical Research and the University of North Carolina-Chapel Hill Center for Health Equity Research [32]. While every effort was made to avoid duplication in CDEs, some further harmonization among the various COVID-19 CDEs may be needed.

### External Expert Review

The broad array of complementary perspectives and expertise among the two groups from the University of Pittsburgh and CONNECTS was essential to making rapid progress towards this first iteration of the NHLBI COVID-19 CDEs. We attempted to mitigate potential bias imparted by the composition of these teams by incorporating multidisciplinary expert appraisal from leaders within the PETAL, SIREN, and GCS-NeuroCOVID networks. Additionally, the VUMC Scientific Committee, VUMC Evidence Synthesis team, and the CONNECTS Steering Committee reviewed and approved of the CDEs. We acknowledge that bias was imparted by the feedback loop we developed with harmonized studies and CONNECTS stakeholders. For example, some of our CDEs were designed with specific studies or interventions and analyses in mind. We are now reworking several CDEs in Version 1.3 to be more inclusive. Conversely, that same feedback loop was also critical to our successful real-time harmonization and implementation efforts. Through this feedback loop, we adapted our CDEs as COVID-19 knowledge changed, and we accommodated nontraditional study designs that became essential for rapid COVID research (e.g., adaptive multitherapeutic designs, no-touch studies).

### Dynamic Development and Versioning

The pace of knowledge generation during the pandemic often informed changes to the data elements we created and prompted us to develop new domains for additional data elements, such as those surrounding vaccination. The NHLBI COVID-19 CDEs and data dictionary also had to be flexible to accommodate competing opinions among the research community on the relative importance of different trial designs and therapeutics, while still creating targeted and functional data elements that could facilitate data capture and harmonized analysis for all. Harmonizing each study dataset required hands-on collaboration between our team and each study team wherein every variable and response option was discussed to devise mapping solutions where needed. Mapping to the CDEs was relatively high for subject areas we would expect to see regardless of the type of study (Adverse Events, Disposition, Intervention Exposure, and Healthcare Outcomes). We noted high variability in CDE mapping for the Demographics, Medical History, Organ Support, Disposition, Healthcare Outcomes, and Symptom Burdon domains. In many cases, this variability was influenced by the study population and type of study being conducted. For example, we would expect more detailed mapping of daily vital signs and organ support in inpatient studies than in outpatient studies. Additionally, each of these domains was designed to be expansive, with numerous variables tiered as “optional” priority level included in each domain. These domains were purposefully structured to be flexible such that if a particular variable could be of interest to a future study, it would be captured in a standardized way. Given the pace at which COVID-19 studies were implemented in parallel with CDE development and early understanding of the nature of COVID-19, these differences in mapping levels across studies are to be expected. We note that revisions made in response to study teams’ feedback also considered several factors, including alignment with existing standards, clinical and coordinator feedback, and impact on harmonization efforts. Each version of the NHLBI COVID-19 CDEs is archived so that changes are transparent and accounted for, ensuring that harmonization and mapping efforts are consistent across versions.

### Lessons Learned for CDE Development

The urgency of the pandemic necessitated a “learning while doing” approach, and so our iterative methodology differed from approaches previously described by NCI and NINDS [3,33]. The inclusion of elements from other COVID-19 trials and extant CDE resources in the NHLBI COVID-19 CDEs helped facilitate interoperability and reuse of ongoing and future COVID-19 studies, as advocated in the FAIR principles. Based on our experiences, we recommend using CDISC as the foundational data standard for future COVID-19 trials. CDISC provided the flexible yet targeted balance we needed to harmonize CONNECTS studies, which used disparate elements for data collection as a result of the unprecedented rate of initiation of COVID-19 clinical trials and the intensive time needed to develop CDEs. At this point of the pandemic, trialists are in a better position to adopt COVID-19 CDEs from the beginning of their study as a new phase of COVID-19 research begins.

Historically, CDEs often fail to get adopted widely because there is little incentive for researchers to make the effort to tailor their data collection for re-use [34], and studies often need to balance between CDE adoption and data collection burden. The promotion of data sharing has seen multiple CDE development efforts in recent years. There is a top-down approach at the NIH to develop CDEs for research supported by each Institute instead of a particular study or consortium, as listed in the NIH CDE Repository (https://cde.nlm.nih.gov/cde/search), as well as the bottom-up approach for a specific Consortium [35-37]. The top-down approach of CDE development has the benefit of broad coverage and adoption, but adoption at the study or consortium level can be challenging. On the contrary, the bottom-up approach has better consortium-specific adoption but lacks wider adoption outside of the consortium. We tried to realize the benefits of both approaches to serve our consortium, and beyond, by engaging with COVID-19 Trials outside of CONNECTS and with the broader community by submitting to the NIH CDE Repository (in progress). There are several factors that contributed to our effective CDE development and implementation: 1) Early consortium recognition and support for data sharing that was explicitly written into study data sharing milestones and supported by a dedicated DHC for CDE development, CDE adoption, and harmonization; 2) A multidisciplinary team with complementary expertise and experience; 3) Our adherence to guiding principles methodology at the outset; and 4) Dynamic interaction with study teams for near instantaneous feedback for both *post hoc* harmonization and mapping as well as during study design and development.

Despite our intentionality in creating flexible CDEs to support broad adoption and the support from NHLBI and CONNECTS, we experienced some issues. We spent considerable time discussing CDEs that were not critical to the success of a COVID-19 study. For example, the demographics domain was discussed at length, but less than half of its CDEs are core. If this CDE process were repeated, we recommend focusing on standard demographic variables (Age, Sex, Race, and Ethnicity). If other subject characteristics are of interest, we recommend selecting a single assessment for question and response, and not attempting to refine the existing assessments. Our custom COVID-19 CDEs attempted to serve specific research questions, which were still in flux at the time of CDE creation. In retrospect, the CDEs may have been more robust and dynamic to the changing COVID-19 landscape if we focused on events or conditions, instead of the research question itself. For example, in upcoming CDE version 1.3, we revised the vaccine domain to collect data about vaccinations, instead of a questionnaire summarizing a participant’s vaccine experience. Although it was impossible to predict in 2020 what a participant’s COVID-19 vaccine experience would look like, the event-style domain worked just as well for one dose as it will for multiple doses. Lastly, our CDEs were designed to guide both raw data capture and harmonized data submission. By accomplishing both, sacrifices were made to each individual objective. For example, some CDE questions and controlled responses were not ideal for self-administered assessments. Likewise, CDEs optimized for data collection schedules may not lend to discussion or analysis beyond the data collection schedule.

### Future Developments

Data standardization for COVID-19 trials was the major objective of creating the set of NHLBI COVID-19 CDEs. However, it is possible that some data elements and domains could be applicable to other diseases and conditions, and we considered the potential for reuse when developing the CDEs. For example, we were unable to identify many existing CDEs for interventional studies of acute respiratory infections; our work could be of use for other respiratory infections or conditions beyond COVID-19. We will continue to pursue additional dissemination efforts, including upload to the NIH CDE Repository, as well as testing the effectiveness of our harmonization efforts through cross-study analysis of CONNECTS harmonized data.

## Conclusion

We have developed a set of living COVID-19 CDEs that are being maintained to reflect advances in knowledge and research regarding COVID-19 disease. Investigators can incorporate these CDEs in their entirety or *à la carte*, depending on what is most germane to their study’s intervention, population, or design. At a minimum, we encourage use of the core set of data elements in both inpatient and outpatient, non-interventional and interventional, COVID-19 studies to standardize data collection and allow for trial comparisons and data pooling in the future.

## Supplementary material

For supplementary material accompanying this paper visit https://doi.org/10.1017/cts.2022.466. click here to view supplementary material
