# Supplementary material for: Development and implementation of the National Heart, Lung, and Blood Institute COVID-19 common data elements
Source: J Clin Transl Sci. 2022 Sep 26;6(1):e142. doi: 10.1017/cts.2022.466 (PMC9794959; doi:10.1017/cts.2022.466)
Supplement: Supplementary file 1 [file S2059866122004666sup001.docx]

**SUPPLEMENTARY MATERIAL**

NHLBI COVID-19 CDEs, CONNECTS CDE Manual, and Data Dictionary (<https://nhlbi-connects.org/common_data_elements>)

**Supplementary Table 1. Timeline of CDE versioning and revisions from conception to Version 1.0 Release**

**Supplementary Table 2. Summary of precedents that informed initial CDE development**

| **Studies and Established Data Collection Precedents** |
| --- |
| Repository of Electronic Data COVID-19 Observational Study (RED CORAL)^a^ |
| Biology and Longitudinal Epidemiology of PETAL COVID-19 Observational Study (BLUE CORAL)^a^ |
| Outcomes Related to COVID-19 Treated with Hydroxychloroquine among In-patients with Symptomatic Disease (ORCHID)^a,b^ |
| Solidarity Trial for COVID-19 Treatments (Solidarity)^a^ |
| Viral Infection and Respiratory Illness Universal Study (VIRUS)^a^ |
| Randomized, Embedded, Multi-factorial, Adaptive Platform (REMAP)-COVID^a^ |
| International Severe Acute Respiratory and emerging Infection Consortium (ISARIC)^a^ |
| Convalescent Plasma in Outpatients with COVID-19 (C3PO)^a,b^ |
| Accelerating COVID-19 Therapeutic Interventions and Vaccines (ACTIV)-3^a,b^ |
| Randomised Evaluation of COVID-19 Therapy (RECOVERY)^a^ |
| Trial of Treatments for COVID-19 in Hospitalized Adults (DisCoVeRy)^a^ |
| Trial of Early Therapies During Non-hospitalized Outpatient Window for COVID-19 (TREAT-NOW)^b^ |
| Passive Immunity Trial for Our Nation to Treat COVID-19 in Hospitalized Adults (PassItOn)^b^ |
| PhenX Toolkit^b^ |
| *All of Us* Research Program^b^ |
| Clinical Data Acquisition Standards Harmonization (CDASH)^b^ |
| Common Terminology Criteria for Adverse Events (CTCAE)^a^ |
| NIH CDE Repository^a^ |

1. Precedents initially identified by the University of Pittsburgh CDE team
2. Precedents initially identified by the CONNECTS Data Harmonization Core

**Supplementary Table 3. Data dictionary structure example for the Organ Support Domain**

**Supplementary** **Figure 1. Overview of the evolution of the NHLBI COVID-19 CDE versions**


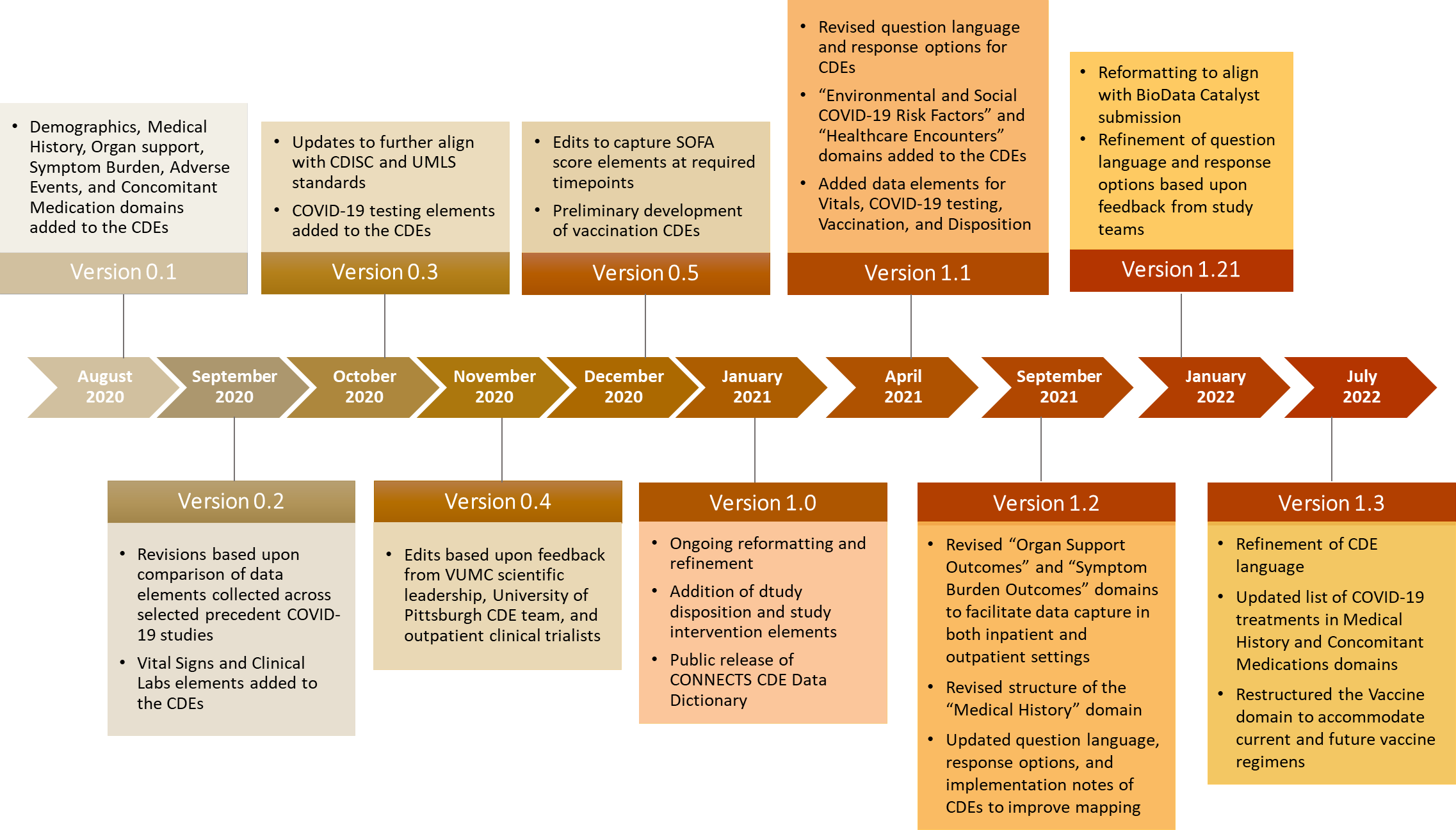


**Supplementary Figure 2. BioData Catalyst submission activities for CONNECTS Studies**


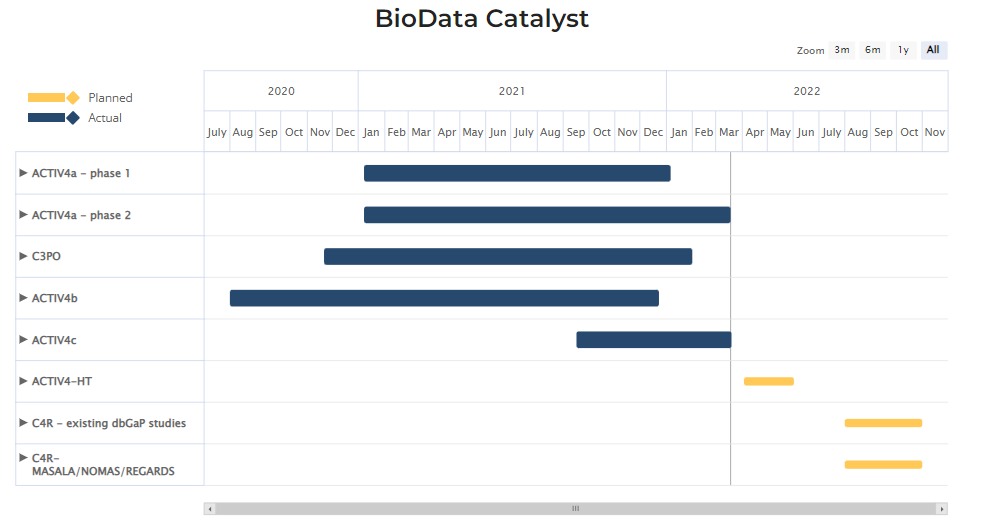


**Supplementary Figure 3. Proportion of NHLBI COVID-19 CDEs mapped to ACTIV-4a study variables**

**Supplementary Figure 4. Proportion of NHLBI COVID-19 CDEs mapped to ACTIV-4b study variables**

**Supplementary Figure 5. Proportion of NHLBI COVID-19 CDEs mapped to C3PO study variables**

**Supplementary Figure 6. Proportion of NHLBI COVID-19 CDEs mapped according to study type across the ACTIV-4a, ACTIV-4b, and C3PO studies**
